# Determinants of heterosexual men's demand for long-acting injectable pre-exposure prophylaxis (PrEP) for HIV in urban South Africa

**DOI:** 10.1186/s12889-019-7276-1

**Published:** 2019-07-24

**Authors:** Chih-Yuan Cheng, Matthew Quaife, Robyn Eakle, Maria A. Cabrera Escobar, Peter Vickerman, Fern Terris-Prestholt

**Affiliations:** 1 Faculty of Public Health and Policy, London School of Hygiene and Tropical Medicine, 15-17 Tavistock Place, London, WC1H 9SH UK; 2 Medical Faculty Mannheim, Heidelberg University, Theodor-Kutzer-Ufer 1-3, 68167 Mannheim, Germany; 3 Division of Health Economics, German Cancer Research Center (DKFZ), Im Neuenheimer Feld 280, 69120 Heidelberg, Germany; 4 Wits RHI, University of the Witwatersrand, 22 Esselen Street, Hillbrow, Johannesburg, 2001 South Africa; 5 School of Social and Community Medicine, University of Bristol, Canynge Hall, 39 Whatley Road, Bristol, BS8 2PS UK

**Keywords:** HIV prevention, Long-acting injectable pre-exposure prophylaxis, Heterosexual men, South Africa, Biomedical strategies

## Abstract

**Background:**

Heterosexual men in South Africa are a large key population to exposure to HIV, yet preferences for HIV pre-exposure prophylaxis (PrEP) among this population have not, to date, been investigated in the literature. This paper aims to explore HIV prevention preferences among heterosexual men in urban South Africa, as well as to examine the demand and characteristics of men who favour long-acting injectable (LAI) PrEP over condoms and oral PrEP.

**Methods:**

Data were collected among 178 self-reported HIV-negative heterosexual men, who were given example products and information before being asked which they preferred. Multivariate logistic regression was used to analyse which characteristics were associated with product choice.

**Results:**

48% (*n* = 85) of participants preferred LAI PrEP, while 33% (*n* = 58) and 20% (*n* = 35) chose oral PrEP and condoms respectively. Having children (marginal effect = 0.22; 95% CI [0.01, 0.44]) or having higher risk attitude scores (marginal effect = 0.03; 95% CI [0.01, 0.06]) was significantly associated with a choice of LAI PrEP, while those who had unprotected anal intercourse (marginal effect = − 0.42; 95% CI [− 0.57, − 0.27]) and those who were concerned with protection against other sexually transmitted infections over HIV (marginal effect = − 0.42; 95% CI [− 0.60, − 0.24]) appeared less likely to prefer LAI PrEP.

**Conclusions:**

The results suggested a relatively high demand and theoretical acceptability for LAI PrEP among heterosexual men in urban South Africa, but there appeared to be fewer distinct predictors for the willingness to use LAI PrEP compared to studies conducted among gay and bisexual men and women. Nevertheless, the findings contribute to the mapping of the demand and determinants of heterosexual men’s preferences for novel antiretroviral-based prevention in sub-Saharan Africa, and the data could aid in the differentiated design of future HIV prevention strategies using LAI PrEP in conjunction with other methods.

**Electronic supplementary material:**

The online version of this article (10.1186/s12889-019-7276-1) contains supplementary material, which is available to authorized users.

## Background

South Africa has the highest number of people living with HIV, which was 7.1 million in 2016 [1]. The high prevalence is partly attributable to the decrease in AIDS mortality and the increase in life expectancy [2] due to a relatively high HIV testing rate [3] and access to antiretroviral treatment (ART), which increased from 19% in 2010 to 56% in 2016 [1]. Despite efforts to improve HIV testing and treatment coverage, in 2016 there were an estimated 260,000 incident HIV cases among adults (aged 15 and over) in South Africa, an annual incidence of 1% [1]. The high rate of new infections possibly results from multiple factors – biological, socio-behavioural and socio-economic [4]. According to the South African National HIV Survey [5], the main route of transmission is through heterosexual sex, which suggests that alongside the needs of focusing on key populations such as female sex workers and men who have sex with men, the heterosexual population should also be a focal point for effective HIV prevention methods.

There are various HIV prevention products currently being used or developed. Condoms have been a long-standing and efficacious method to prevent HIV transmission and other sexually transmitted infections (STIs) [6], yet consistent condom usage is low in the general population – only 31.9% of the general population aged over 15 reported using condoms every time or almost every time with their last sexual partner in the South African National HIV Survey in 2012 [5].

Oral pre-exposure prophylaxis (PrEP) antiretroviral (ARV) agents have been proven efficacious in clinical trials at preventing HIV acquisition – efficacy ranged from 44 to 75% reduction in HIV acquisition in heterosexual men and women, men who have sex with men and people who inject drugs [7]. The regimen most commonly used is daily oral emtricitabine plus tenofovir (FTC/TDF) with regular HIV tests and blood tests to monitor for adverse effects [8]. Both oral and topical PrEP products have been more effective in male populations than female [9, 10], partly explained by adherence, and partly by pharmacokinetic data indicating higher colorectal drug concentrations than in the female lower genital tract [11, 12]. As of May 2018, oral PrEP was provided to female sex workers, men who have sex with men, and young people at university and community testing sites in South Africa [13, 14]. Uptake rates for female sex workers and men who have sex with men, as of the end of 2017, were 12 and 47%, respectively. Uptake rate for young people was not yet available [15]. Altogether, there were an estimated 16,000 oral PrEP users in South Africa [16].

Long-acting injectable (LAI) PrEP has the potential to be efficacious, and should only require an injection every two to three months [17], which may mitigate the adherence issues of oral PrEP [18, 19]. The phase II safety trials for long-acting cabotegravir (CAB LA) and long-acting rilpivirine (RPV LA) have shown both to be safe and tolerable [20, 21]. The results of CAB LA phase III efficacy trials (HPTN 083 and 084) focusing on the populations of men who have sex with men, transgender women, and women at high risk of acquiring HIV infection are expected to follow in 2022 [22, 23]. With the addition of this potential new ARV-based prevention method, it expands the “PrEP method mix” for a wider user choice and is expected to increase the acceptability and uptake of HIV prevention measures [24].

For HIV prevention resources to be deployed as effectively as possible, it is essential to identify who among potential users might be interested in using different products. Predominant attention has been paid to key population groups for HIV transmission, including but not restricted to men who have sex with men, people who inject drugs, male and female sex workers and adolescent girls and young women [25–27]. Despite 75% of new infections in Eastern and Southern Africa being among heterosexual men and women [28] and broad roll-out of interventions including voluntary medical male circumcision, there has been little focus on how ARV-based prevention could enhance HIV prevention programmes among heterosexual men.

To make a new treatment or prevention measure accessible, users’ adoption is one of the essential facets. To facilitate the adoption by potential users, it entails an understanding of their needs and preferences for the new technology early in the development phase [29], as is commonly done in pre-marketing research of other consumer products [30]. Given that men in sub-Saharan Africa were shown to be less likely to engage in the HIV testing and ART treatment cascade compared with women [31, 32], it is critical to understand the perception of men towards a novel ARV-based prevention option during its development, so as to ensure the product meets their needs and they will thus use.

There is a rich literature on the willingness to use oral PrEP in both heterosexual men and men who have sex with men in low- and middle-income countries (LMIC) [25, 33–35]; on the other hand, studies investigating men’s willingness to use LAI PrEP have predominantly focused on men who have sex with men in high-income settings. In a study by Meyers et al. [36], 80% of young men who have sex with men in New York City expressed interest in using LAI-PrEP, and Parsons et al. [37] found 46% of gay and bisexual men preferred LAI PrEP compared to oral PrEP. The commonly mentioned reasons for preferring LAI PrEP were convenience and longer protection duration [38]. However, there is yet no study exploring the preferences of heterosexual men for LAI PrEP in LMIC settings.

With a need to understand an important user group’s demand and perception towards a novel HIV prevention measure early in the development phase and to fill the gap in the literature, we used a multivariate logistic regression model to analyse the characteristics of heterosexual men preferring LAI PrEP in South Africa. To the best of our knowledge, this is the first study exploring the demand and preference towards LAI PrEP among sub-Saharan heterosexual men. This paper contributes to the literature by differentiating heterosexual men who favour LAI PrEP from others preferring oral PrEP and condoms, as well as presenting data which could inform strategies for the future roll-out and the design of more focused HIV prevention schemes for heterosexual men in sub-Saharan Africa.

## Methods

### Data

Secondary data analysis was conducted on survey data from a study on preferences for new HIV-prevention technologies in urban South Africa in 2015. A detailed protocol of the study design, sample selection and survey methods is presented elsewhere [39, 40]. In brief, data were collected through a population-based household survey among 202 adult men. The township of Vosloorus in Ekurhuleni Metropolitan Municipality, near Johannesburg, was selected for the surveys, given the broad range of demographic, socio-economic, and cultural characteristics within the residential composition [41]. Ethical approval for the survey was obtained from both London School of Hygiene and Tropical Medicine (Ref no. 8451–2) and University of Witwatersrand Human Research Ethics Committee (Ref no. M140614), and informed consent was obtained from all participants.

Of the 202 men sampled, 178 men self-reported as HIV-negative and heterosexual. There were three choices of HIV prevention products in the survey which the male participants ranked from most to least preferred. The three options were LAI PrEP, oral PrEP, and condoms. Participants were asked to rank products after being given information on each (see Additional file 1**:** Appendix 1), an opportunity to hold example products, and the chance to ask fieldworkers any questions they had.

### Determinants for demanding LAI PrEP

There is limited literature probing the willingness of heterosexual men to use LAI PrEP, and we therefore reviewed studies assessing determinants of willingness to use LAI or oral PrEP in any population group. The core literature we followed included a multinational study by Eisingerich et al. [25] and studies by Meyers et al. [36] and Parsons et al. [37], with determinants falling into two categories: socio-demographic factors and risk profile. From a pilot study exploring attributes on HIV products valued by potential users [39, 41], five key attributes were identified. These attributes were also included in this study to examine if they were factors influencing the willingness to use LAI PrEP in heterosexual men. Based on the literature, we predicted a priori direction of influence of each variable on the probability of choosing LAI PrEP. The full list of variables and the predicted direction of influence can be found in Table 1.

**Table 1:** Predicted direction of influence, descriptive statistics of independent variables and results of bivariate analysis

	Predicted influence on the preference for LAI PrEP^a^	Full sample (*N* = 178)	Preference for HIV prevention products		χ^2^ or coefficient of univariate logistic regression, *P*-value
			LAI PrEP (N = 85)	Oral PrEP or condoms (*N* = 93)	
		N (%) or *(median, range)*	N (%) or *(median, range)*	N (%) or *(median, range)*	
Socio-demographic factors					
Age (median, range)	–	*27 (18–45)*	*28 (18–45)*	*27 (18–45)*	0.000^b^, 0.983
Education					0.008, 0.928
Lower than high school	–	144 (80.9)	69 (81.2)	75 (80.4)	
Finished high school	+	34 (19.1)	16 (18.8)	18 (19.6)	
Household income > R5000	+/−	49 (27.5)	25 (29.4)	24 (25.8)	0.289, 0.591
Have children	–	91 (51.1)	47 (55.3)	44 (47.3)	1.132, 0.287
Median No. of children (range)		*1 (0–7)*	*1 (0–7)*	*0 (0–4)*	0.040^b^, 0.158
Risk Profile					
Self-reported HIV status					1.943, 0.163
Tested, negative	+	136 (76.4)	61 (71.7)	75 (80.6)	
Not-tested	–	42 (23.6)	24 (28.3)	18 (19.4)	
More than 1 sexual partner in the last year	+	79 (44.4)	40 (47.1)	39 (41.9)	0.472, 0.492
Median No. of sexual partners in the last year (range)		*1 (0–50)*	*1 (0–50)*	*1 (0–25)*	0.002^b^, 0.849
Condom usage					0.086, 0.769
Always	+	90 (50.6)	42 (49.4)	48 (51.6)	
Inconsistent	–	88 (49.4)	43 (50.6)	45 (48.4)	
Ever had UAI	+	10 (6)	1 (1.2)	9 (9.7)	6.053, 0.014
Median score for risk attitude (scale 1 = risk-averse; scale 10 = risk-loving; range)	+/−	*7 (1–10)*	*7 (1–10)*	*5 (1–10)*	0.028^b^, 0.016
Product Attributes					
Effectiveness	+/−	148 (83.1)	70 (82.4)	78 (83.9)	0.073, 0.787
Contraception	–	10 (5.6)	6 (7.1)	4 (4.3)	0.637, 0.425
Use frequency	+	6 (3.4)	4 (4.7)	2 (2.2)	0.890, 0.345
STIs prevention	–	12 (6.7)	4 (4.7)	8 (8.6)	1.072, 0.300
Side effects	–	7 (4)	2 (2.4)	5 (5.4)	1.075, 0.300

*UAI* Unprotected anal intercourse, *STIs* Sexually transmitted infections

a. “+” denotes positive influence; “-” denotes negative influence

b. Used univariate logistic regression

#### Socio-demographic factors

The literature suggested that younger participants [25, 37], higher educated [36–38] and with fewer children [25] were more likely to choose LAI PrEP. Therefore, we predicted ‘age’ and ‘have children’ to have a negative influence on the probability for participants to choose LAI PrEP, whereas ‘finished high school education’ has a positive impact. Income level was not found to be a determinant affecting the willingness to use LAI PrEP in the literature [36, 37]. In our study, we defined a monthly income exceeding ZAR 5000 (USD$ 380) as the higher-income group, which accounted for the upper 28% of income level in this study population.

#### Risk profile

According to the Health Belief Model [42], individuals’ perception of their own likelihood of susceptibility to an illness plays a part in influencing their readiness to take actions in health-related behaviour. Thus, those who believe they are more susceptible to contracting HIV might be more likely to take preventive measures, which supports the findings in previous literature [25, 36, 37]. Previously tested for HIV [25], frequent condom use [25], greater number of sexual partners [36] and engaging in unprotected anal intercourse (UAI) [37] were identified as positive predictors for people who are more willing to use LAI PrEP from the limited existing literature, and we expected to see the same direction of impact of these variables on the willingness to uptake LAI PrEP in our study. Being married or in a steady relationship might also serve as a proxy for the risk profile of heterosexual men. However, literature from South Africa showed that the relationship between HIV and marital status is complex [5, 43, 44], and sexual behaviour might act as an intervening variable between them [43]. Additionally, we tested our regression model with and without the relationship status, and it did not change the results. Therefore, with the inclusion of variables representing risky sexual behaviour, we did not include relationship status as a determinant.

We also asked the participants to assess their own risk attitudes by choosing a number on a scale ranging from 1 to 10, whereby 1 was the most risk-averse and 10 was the most risk-loving. Following the Health Belief Model, the more risk-loving the participants were, the more likely they would use preventive products. However, we were uncertain about which product the participants would choose.

#### Product attributes

In the survey, participants were asked to rank five key pre-defined attributes in the order of importance when considering an HIV prevention product: how much it protects one from HIV (‘effectiveness’); whether it can prevent pregnancy (‘contraception’); how often one has to use it (‘use frequency’); whether it protects people from sexually transmitted infections (‘STI prevention’); and what side effects it might have (‘side effects’). Questions asked in the survey can be found in (Additional file 1**:** Appendix 2**).**

Eighty-three percent of the participants in the study by Meyers et al. [36] expressed their concern about the effectiveness of LAI PrEP. Marra & Hankins [26] also demonstrated that the effectiveness of oral PrEP was one of the important factors in a survey for the perception and willingness to use oral PrEP among men who have sex with men in the Netherlands. Given the effectiveness of HIV prevention products tends to be a top concern for users, we did not anticipate people who chose LAI PrEP to perceive product effectiveness differently from those who chose other methods. Previous clinical trials have shown that low adherence compromises the efficacy of oral PrEP, and having to take oral PrEP daily may confer the difficulty that reduces adherence [45]. Participants in a US study also expressed convenience and longer protection duration being the reasons for choosing LAI PrEP [38]. Therefore, we expected people concerned about ‘use frequency’ would be more willing to use LAI PrEP. Several studies probing the willingness to use oral PrEP revealed concerns over potential adverse effects [26, 46]; hence, we anticipated that men in this study would have the same concern regarding side effects when considering a newer product, LAI PrEP. Lastly, as LAI PrEP is not able to prevent pregnancy and STIs, we predicted ‘contraception’ and ‘STI prevention’ to have a negative influence on the likelihood of choosing LAI PrEP.

### Statistical methods

A dichotomous outcome variable was created – with 1 for LAI PrEP being the respondents’ top preferred product and 0 if a condom or oral PrEP were preferred. We first performed a bivariate analysis using Chi-square test to test independent associations between outcomes and the predictors (socio-demographic factors, risk profiles and product attributes); for the associations between outcomes and predictors which are continuous variables (age and risk attitude scores), we used univariate logistic regression. We then conducted a multivariate analysis by applying logistic regression to analyse determinants of ranking LAI PrEP over the other two products with the same set of predictors. An alpha level of 0.05 was used. We also tested variance inflation factors (VIF) to examine multicollinearity among the variables. The analysis was conducted in Stata/SE 13.1.

Survey weighting was applied to the multivariate regression. The participants were divided into two groups according to the standard cut-off age of young adults [47]: under 25 years old and aged 25 or above. The proportion of these two age groups was compared with the proportion of the total South African male population. Weights were given to the two age groups in our survey sample to match the proportion in the general population with the aim that our results can be generalised to other provinces in South Africa.

## Results

### Descriptive statistics and bivariate analysis

The descriptive statistics are presented in Table 1. Among the three HIV prevention methods, 48% (*n* = 85) of participants chose LAI PrEP with 33% (*n* = 58) and 20% (*n* = 35) choosing oral PrEP and condoms, respectively. Even though participants were told LAI PrEP was not available yet, it was the most popular HIV prevention methods in this survey.

The overall median age of the study population was 27 years old (range = 18–45, interquartile range = 23–35). Twenty-four percent (*n* = 42) of the participants in this study had never received an HIV test. Forty-four percent (*n* = 79) of men in this study reported having more than one sexual partner in the past 12 months, and the median number of sexual partners was one. Forty-nine percent (*n* = 88) of the participants had unprotected sex intermittently, and 6 % (*n* = 10) of the study population experienced UAI. The median score for the risk attitude scale was 7, and 56% (*n* = 100) of the participants considered themselves to be risk-loving, where they rated themselves 6 out of 10 or above on the scale. As for the five product attributes, 83% (*n* = 148) of men regarded “efficacy” of the products as the most important attribute when they considered which product to choose, whereas each of the rest of four attributes was ranked most important by fewer than 10% of participants respectively.

The bivariate analysis did not reveal independent associations between each predictor and the preference for LAI PrEP – the only exceptions being the experience of having UAI (χ^2^ = 6.05, *P* = 0.01) and having a higher risk-attitude score (coefficient = 0.03, *P* = 0.02). In an additional bivariate analysis between groups (LAI PrEP vs. oral PrEP and LAI PrEP vs. condoms), the determinants “had UAI” and “with a higher risk-attitude score” persisted in showing significant differences between men in favour of LAI PrEP and those preferring condoms. The consideration of each product attribute remained insignificant. (See Additional file 1: Table S1).

### Multivariate analysis

The results are presented as marginal effects in Table 2. VIFs for all variables were below 10, indicating multicollinearity is not an issue in this model.

**Table 2:** Marginal effects of multivariate logistic regression: probability of choosing LAI PrEP as preferred product

	Coefficient	95% CI	*P*-value
Socio-demographic factors			
Age	− 0.004	− 0.018, 0.010	0.581
Education			
Lower than high school (as reference)	–	–	–
Finished high school	0.007	−0.248, 0.263	0.954
Household income > R5000	0.024	−0.182, 0.231	0.818
Have children	0.224	0.012, 0.435	0.038
Risk Profile			
Tested HIV before	0.106	−0.094, 0.306	0.300
Multiple sexual partners in the last year	0.008	−0.167, 0.183	0.930
Condom usage			
Always	0.073	−0.109, 0.255	0.433
Inconsistent (as reference)	–	–	–
Ever had UAI	−0.420	−0.574, − 0.265	0.000
Score for risk attitude (scale 1 = risk-averse; scale 10 = risk-loving)	0.034	0.006, 0.063	0.017
Product Attributes			
Effectiveness	−0.293	−0.679, 0.093	0.137
Contraception	−0.081	−0.632, 0.470	0.773
Use frequency	−0.016	−0.506, 0.473	0.949
STIs prevention	−0.417	−0.596, − 0.237	0.000
Side effects	−0.316	− 0.651, 0.019	0.065

*UAI* Unprotected anal intercourse, *STIs* Sexually transmitted infections

#### Socio-demographic factors

Men who have children were associated with a higher likelihood of choosing LAI PrEP relative to childless men, where the likelihood increased by 22% (marginal effect = 0.22; 95% CI [0.01, 0.44]). Other variables in this category did not reach statistical significance.

#### Risk profile

Somewhat surprisingly, men who have ever had UAI were associated with a decreased probability of 42% (marginal effect = − 0.42; 95% CI [− 0.57, − 0.27]) of choosing LAI PrEP compared to those who never had a UAI. Regarding the self-rated risk attitude score, the increase of one point on the risk scale was associated with a 3% (marginal effect = 0.03; 95% CI [0.01, 0.06]) increased likelihood of choosing LAI PrEP, which suggests that a risk attitude score of 10 predicts roughly 30% higher probability of choosing LAI PrEP compared to the most risk-averse (with score 1). No association was found between the choice of LAI PrEP with having been previously tested for HIV, frequent condom use or having multiple sexual partners.

#### Product attributes

Respondents who valued the provision of STI prevention the most were associated with 42% (marginal effect = − 0.42; 95% CI [− 0.60, − 0.24]) less likely to choose LAI PrEP. The preference for the rest of the product attributes did not show significant association on the choice of LAI PrEP.

## Discussion

The aim of this study was to investigate the HIV prevention preferences among heterosexual men, as well as to examine the demand and a priori determinants of men who favour LAI PrEP in particular over other products, in order to contribute to the notably limited literature exploring the acceptability of ARV-based HIV prevention products among heterosexual men in LMICs.

Almost half of the participants in this study indicated a preference for LAI PrEP over oral PrEP or condoms, with the effectiveness of the HIV prevention method being the most important product attribute. Bivariate analysis showed the independent associations between the preference for LAI PrEP and experience of unprotected anal intercourse as well as risk-loving attitude. The results of multivariate logistic regression highlighted that men who have children and who scored higher risk attitude scale were more likely to choose LAI PrEP as their preferred HIV prevention method. On the contrary, men in this study were more likely to choose the other two HIV prevention products, oral PrEP or male condoms, if they valued whether the products prevented other STIs, or if they ever had unprotected anal intercourse.

Men who are more risk-loving (with higher scores in the risk attitude scale) were shown to be associated with a higher likelihood to demand LAI PrEP. To our knowledge, no literature explored the relationship between risk attitude and the preference of HIV prevention products. However, studies have shown some risky sexual behaviours, e.g., multiple sexual partners [36] and unprotected anal sex with casual partners [37], are associated with preference for LAI PrEP. This might indirectly indicate the association between risk-loving attitude and the preference for LAI PrEP, but it still warrants further study. It is also plausible that those who perceived the ability to prevent other STIs as the most important attribute for an HIV prevention product may not take up LAI PrEP, given LAI PrEP does not have this attribute.

Two statistically significant determinants, ‘having children’ and ‘ever had UAI,’ influenced the likelihood of choosing LAI PrEP in the opposite direction as we predicted. Having children was identified as a determinant that would increase the likelihood of choosing LAI PrEP in our study, whereas the opposite was found by Eisingerich et al. [25] However, the populations in the survey by Eisingerich et al. who were likely to have children were young women, female sex workers, and serodiscordant couples [25]. Little literature was found to discuss the influence of the number of children on the willingness of using either LAI PrEP or oral PrEP in any of these groups. Therefore, it is unclear that the inconsistent findings in our study and the literature were due to the differences between males and females, which would require future study to explore.

Parsons et al. [37] revealed that gay and bisexual men who had UAI in the previous three months were associated with the preference for LAI PrEP. In contrast to heterosexual men, a higher proportion of gay and bisexual men would engage in unprotected receptive anal intercourse (URAI), which was found to be associated with higher uptake of oral PrEP in a cohort study on gay and bisexual men [48]. Literature suggested that the per-partner HIV transmission risk is double for URAI compared to unprotected insertive anal intercourse (UIAI), at 40 and 22% respectively [49]. Following the Health Belief Model, given the higher risk of URAI, gay and bisexual men might have a higher demand for more effective HIV prevention methods than heterosexual men (mostly engaged in UIAI), which provides a potential explanation for why we did not find UAI as a positive predictor for choosing LAI PrEP in our study.

Among studies exploring the predictors for the preference for LAI PrEP, Meyers et al. [36] discovered that men who have sex with men in New York City, who had a history of STIs and multiple sexual partners in the previous three months, had a higher willingness to use LAI PrEP, whereas people with higher socioeconomic status and a college degree had a lower willingness. Two US studies in men who have sex with men also revealed that age and higher education attainment were positively associated with the preference for LAI PrEP, and the average age for people who favour LAI PrEP was 40 and 24 years old, respectively [37, 50]. A multinational study on female sex workers, men who have sex with men, serodiscordant couples, people who inject drugs, and young women also found that people who had been tested for HIV and those with frequent condom use might prefer oral PrEP [25]. In our study, age, education, income, previously tested for HIV, having multiple sexual partners, and always using a condom did not demonstrate differences in the preference for LAI PrEP compared to other HIV prevention products. It is not clear whether the discrepancy is due to different populations (men who have sex with men, females and heterosexual men) or different geographical regions and contexts (the US and South Africa), which would also be important to assess in future research.

### Study limitations

There are some limitations in this study. Firstly, all variables came from self-reported data which are inherently subject to response bias, especially when the participants were asked the questions about their risk profile by the interviewer. For example, although the stigma against HIV has improved in the past decade [5], the disclosure of HIV status is still of concern in South African society [5, 51]. Therefore, the self-reported HIV-positive status might be underreported.

Another limitation is that the costs of the three products were not considered in this study. As LAI PrEP is under development, it is unclear if an effective LAI will be developed, or whether it would be sufficiently cost-effective to be made available in LMIC contexts. Some studies have demonstrated that participants are more willing to take up PrEP if it is offered for free at the point of access [46, 52, 53]. Therefore, the financial sustainability of LAI programmes will need to be considered against the need to maximise uptake.

Finally, indicating product preference in a survey may not mean that men are able to initiate and sustain effective use of LAI PrEP, or any preventative product. Future research is needed on how individual, social, and structural factors may influence product uptake and use.

## Conclusions

LAI PrEP has relatively high theoretical acceptability among heterosexual men in urban South Africa, where the effectiveness of products is found to be highly critical to the attractiveness of an HIV prevention product. However, there appeared to be less distinct determinants to predict the willingness of men to take up LAI PrEP compared to the studies conducted among gay and bisexual men and women – the only positive predictors found in this study were having children and being more risk-loving. Heterosexual men are an under-researched but critical population in the HIV response in countries with a generalised HIV epidemic; more research is needed to explore how preferences in this group differ from other key population groups. Nonetheless, the findings from this study suggest there is demand for LAI PrEP in urban heterosexual men in South Africa, and these data can aid in the differentiated design of future HIV prevention strategies using LAI PrEP in conjunction with other methods.

## Additional file

Additional file 1: **Appendix 1.** Product information provided prior to choice of the products. Appendix 2. Questions asked for attributes of the products. **Table S1.** Descriptive statistics of independent variables and results of bivariate analysis by preferences (LAI PrEP, oral PrEP and condoms). (DOCX 30 kb)

## Data Availability

Data would be made available by the corresponding author upon reasonable request.

## Abbreviations

AIDS: acquired immunodeficiency syndrome
ART: antiretroviral therapy
ARV: antiretroviral
CI: confidence interval
HIV: human immunodeficiency virus
LAI PrEP: long-acting injectable pre-exposure prophylaxis
LMIC: low- and middle-income country
PrEP: pre-exposure prophylaxis
STI: sexually transmitted infection
UAI: unprotected anal intercourse
UIAI: unprotected insertive anal intercourse
URAI: unprotected receptive anal intercourse
