# Supplementary material for: Determinants of heterosexual men's demand for long-acting injectable pre-exposure prophylaxis (PrEP) for HIV in urban South Africa
Source: BMC Public Health. 2019 Jul 24;19:996. doi: 10.1186/s12889-019-7276-1 (PMC6657137; doi:10.1186/s12889-019-7276-1)
Supplement: Supplementary file 1 — Appendix 1. Product information provided prior to choice of the products. Appendix 2. Questions asked for attributes of the products. Table S1. Descriptive statistics of independent variables and results of bivariate analysis by preferences (LAI PrEP, oral PrEP and condoms). (DOCX 30 kb) [file 12889_2019_7276_MOESM1_ESM.docx]

#

# Additional file 1

# Appendix 1. Product information provided prior to choice of the products.

**Oral Pre-Exposure Prophylaxis (PrEP) tablets**

(A picture of PrEP tablet was provided here for illustration to the participants.)

Oral PrEP tablets contain a small amount of HIV prevention medicine.

You can use this product without your partner knowing.

Tablets need to be taken more often than other products.

**What happens if I forget to take a tablet?**

Like a contraceptive pill, you can take a tablet up to 24 hours after you missed the dose.

**How long will side effects last?**

Side effects will last for 1-2 hours after you have used the product.

**Can I take the tablets whilst I am pregnant?**

Yes – the tablets are completely safe to you and your baby.

**Where could I get these from?**

These would be available for free from a pharmacy or clinic, or other places contraceptives are available at the moment. None of these products are currently available as they are still being tested.

**The Injection**

(A picture of syringe was provided here for illustration to the participants.)

The injection is the same as any other injections you have had, but it contains a small amount of HIV prevention medicine.

You can use this without your partner knowing.

The injection needs to be used less often than some other products.

**What happens if I forget to get a new injection?**

You will not be protected from HIV or pregnancy.

**How long will side effects last?**

Side effects will last for 1-2 hours after you have used the product.

**Can I get the injection whilst I am pregnant?**

Yes – the gel is completely safe to you and your baby.

**Where could I get these from?**

You would be able to receive an injection from staff at a pharmacy or clinic, or other places contraceptives are available at the moment. None of these products are currently available as they are still being tested.

#

# Appendix 2. Questions asked for attributes of the products.

| Now I would like you to think about what is important to you when considering these products. I am going to read you five things - please rank them in order from the thing you care about most, to the thing you care about least. |
| --- |
| 1. How much it protects you from HIV? |
| 1. Whether it can prevent pregnancy? |
| 1. How often you have to use it? |
| 1. Whether it protects you from other STIs? |
| 1. What side-effects it might have? |

# Additional file 1 Table S1. Descriptive statistics of independent variables and results of bivariate analysis by preferences (LAI PrEP, oral PrEP and condoms)

|  | **Preference for HIV prevention products, full sample (N=178)** | | | **χ^2^ or coefficient of univariate logistic regression, P-value** | |
| --- | --- | --- | --- | --- | --- |
|  | **LAI PrEP (N=85)** | **Oral PrEP (N=58)** | **Condoms (N=35)** | **LAI PrEP vs oral PrEP** | **LAI PrEP vs condoms** |
|  | **N (%) or**  *(median, range)* | **N (%) or**  *(median, range)* | **N (%) or**  *(median, range)* |  |  |
| **Socio-demographic factors** |  |  |  |  |  |
| Age (median, range) | *28 (18-45)* | *26 (18-45)* | *31 (19-45)* | 0.005^a^, 0.389 | -0.007^a^, 0.224 |
| Education |  |  |  | 0.058, 0.810 | 0.252, 0.616 |
| Lower than high school | 69 (81.2) | 48 (82.8) | 27 (77.1) |  |  |
| Finished high school | 16 (18.8) | 10 (17.2) | 8 (22.9) |  |  |
| Household income > R5000 | 25 (29.4) | 16 (27.6) | 8 (22.9) | 0.056, 0.813 | 0.534, 0.465 |
| Have children | 47 (55.3) | 22 (37.9) | 22 (62.9) | 4.163, 0.041 | 0.580, 0.446 |
| Median No. of children (range) | *1 (0-7)* | *0 (0-4)* | *1 (0-4)* | 0.066^a^, 0.054 | -0.001^a^, 0.982 |
| **Risk Profile** |  |  |  |  |  |
| Self-reported HIV status |  |  |  | 0.300, 0.586 | 3.915, 0.048 |
| Tested, negative | 61 (71.7) | 34 (75.8) | 31 (88.6) |  |  |
| Not-tested | 24 (28.3) | 14 (24.1) | 4 (11.4) |  |  |
| More than 1 sexual partner in the last year | 40 (47.1) | 23 (40) | 16 (45.7) | 0.767, 0.381 | 0.018, 0.893 |
| Median No. of sexual partners in the last year (range) | *1 (0-50)* | *1 (0-25)* | *1 (0-22)* | 0.003^a^, 0.724 | -0.001^a^, 0.900 |
| Condom usage |  |  |  | 0.895, 0.344 | 3.677, 0.055 |
| Always | 42 (49.4) | 24 (41.4) | 24 (68.6) |  |  |
| Inconsistent | 43 (50.6) | 34 (48.6) | 11 (31.4) |  |  |
| Ever had UAI | 1 (1.2) | 4 (6.9) | 5 (14.3) | 3.343, 0.067 | 8.970, 0.003 |
| Median score for risk attitude (scale 1=risk-averse; scale 10=risk-loving; range) | *7 (1-10)* | *5.5 (1-10)* | *5 (1-10)* | 0.020^a^, 0.114 | 0.033^a^, 0.008 |
| **Product Attributes** |  |  |  |  |  |
| Effectiveness | 70 (82.4) | 51 (87.9) | 27 (77.1) | 0.434, 0.510 | 0.434, 0.51 |
| Contraception | 6 (7.1) | 2 (18.4) | 2 (5.7) | 0.072, 0.788 | 0.072, 0.788 |
| Use frequency | 4 (4.7) | 1 (13.1) | 1 (2.9) | 0.908, 0.341 | 0.212, 0.645 |
| STIs prevention | 4 (4.7) | 3 (22.3) | 5 (14.3) | 0.016, 0.899 | 3.280, 0.070 |
| Side effects | 2 (2.4) | 4 (25.6) | 1 (2.9) | 1.771, 0.183 | 0.026, 0.872 |

UAI = unprotected anal intercourse; STIs = sexually transmitted infections

a. Used univariate logistic regression
